# Impact of spatial organization on a novel auxotrophic interaction among soil microbes

**DOI:** 10.1038/s41396-018-0095-z

**Published:** 2018-03-23

**Authors:** Xue Jiang, Christian Zerfaß, Song Feng, Ruth Eichmann, Munehiro Asally, Patrick Schäfer, Orkun S Soyer

**Affiliations:** 1 School of Life Sciences, The University of Warwick, Coventry, CV4 7AL UK; 2 Warwick Integrative Synthetic Biology Centre, The University of Warwick, Coventry, CV4 7AL UK; 3 Los Alamos National Laboratory, Theoretical Division (T-6), Center for Nonlinear Studies, Los Alamos, NM 87545 USA

## Abstract

A key prerequisite to achieve a deeper understanding of microbial communities and to engineer synthetic ones is to identify the individual metabolic interactions among key species and how these interactions are affected by different environmental factors. Deciphering the physiological basis of species–species and species–environment interactions in spatially organized environments requires reductionist approaches using ecologically and functionally relevant species. To this end, we focus here on a defined system to study the metabolic interactions in a spatial context among the plant-beneficial endophytic fungus *Serendipita indica*, and the soil-dwelling model bacterium *Bacillus subtilis*. Focusing on the growth dynamics of *S. indica* under defined conditions, we identified an auxotrophy in this organism for thiamine, which is a key co-factor for essential reactions in the central carbon metabolism. We found that *S. indica* growth is restored in thiamine-free media, when co-cultured with *B. subtilis*. The success of this auxotrophic interaction, however, was dependent on the spatial and temporal organization of the system; the beneficial impact of *B. subtilis* was only visible when its inoculation was separated from that of *S. indica* either in time or space. These findings describe a key auxotrophic interaction in the soil among organisms that are shown to be important for plant ecosystem functioning, and point to the potential importance of spatial and temporal organization for the success of auxotrophic interactions. These points can be particularly important for engineering of minimal functional synthetic communities as plant seed treatments and for vertical farming under defined conditions.

## Introduction

Higher-level functions and population dynamics within microbial communities are underpinned by the interactions among the composing species within the community and their environment [1, 2]. Deciphering these interactions is a prerequisite to understand and manage complex natural communities [3] and to achieve community-level synthetic engineering [4–6]. To this end, increasing numbers of experimental studies and (meta)genomic surveys have shown that auxotrophic interactions, involving vitamins and amino acids, are widespread in many microbial natural communities [2, 7–10] and can also be engineered genetically to create synthetic communities [11–13]. Specific auxotrophic interactions among microbes are shown to influence ecosystem functioning; for example, infection outcomes within higher organisms [14], ecological population dynamics in the oceans [2], and the level of biodegradation of organic matter under anoxic conditions [15, 16].

It has been suggested that auxotrophies can result from reduced selective pressures for maintaining biosynthesis capabilities under stable metabolite availability due to abiotic or biotic sources [7, 8]. This proposition is supported by the observed independent evolution of vitamin and amino acid auxotrophies in different, unrelated taxa [17, 18], and points to a direct linkage between ecological dynamics and evolution of auxotrophies [15]. The possible fluctuations in metabolite availabilities in time and space would be expected to impact both the emergence of auxotrophies and the population dynamics of resulting auxotrophic species. For example, in the marine environment, where the observed auxotrophies relate mostly to the loss of biosynthesis capacity for vitamins and amino acids, population dynamics of auxotrophic species are believed to be directly linked to those of “provider” species [2, 7, 19]. The ecological influences of auxotrophic species on community structure and population dynamics can also be exerted by abiotic fluctuations or directly by the abundances and actions of higher organisms within the system.

These ecological influences on microbial population dynamics can increase significantly in spatially organized systems. Yet, the spatial context of microbial interactions is only beginning to be appreciated [10, 20–22]. Considering that each species can display multiple metabolic actions and that all of these can affect a common environment, it is not clear if auxotrophic interactions are always successfully established even if genetic/metabolic complementarity is present. For example, metabolic interactions among genetically engineered auxotrophic yeast are shown to require an initial autonomous growth period to establish [10]. Establishment of these metabolic interactions might also involve spatial factors including the formation of metabolic gradients and specific population organization might be important [15, 20–23]. These spatial factors can include oxygen and pH gradients, as both of these can significantly change upon growth of one species [24–27], and can directly influence subsequent or simultaneous metabolic interactions or growth of different species. Studies exploring the possible interplays between species–species and species–environment interactions have so far used synthetically engineered interactions [10, 20, 21] or enriched microbial communities [15]. Further analyses of such interplay in ecologically and biotechnologically relevant systems can thus help engineering of novel applications of microbial communities with inherent spatial organization, such as seen in agriculture and involving for example closed-ecosystem production, seed treatment, and microbe-based biofertilization [28–30].

Towards this goal, we focus here on identifying potential metabolic interactions among the plant-beneficial endophytic fungus *Serendipita indica* (previously called *Piriformospora indica* [31]) and the ubiquitous soil microbe *Bacillus subtilis*. Identifying a defined media for *S. indica*, we found it to be auxotrophic for the vitamin B1, thiamine. To study the potential auxotrophic interactions of *S. indica*, we then created a co-culture system using *B. subtilis*. We found that *S. indica* thiamine auxotrophy can be satisfied and its growth restored by *B. subtilis*. The success of this auxotrophic interaction, however, is strongly dependent on temporal and spatial organization in the system. These findings and the established synthetic co-culture can act as a basis to develop a more complete functional synthetic community, as advocated for biotechnological applications and for gaining insights into community function [4, 6, 32, 33]. With the inclusion of a plant, such a synthetic community can allow further insights into microbe–microbe, and microbe–plant interactions and development of new agricultural technologies such as in seed coating and vertical farming in controlled environments.

## Results

### *Serendipita indica* is auxotrophic for thiamine

**Fig. 1:**
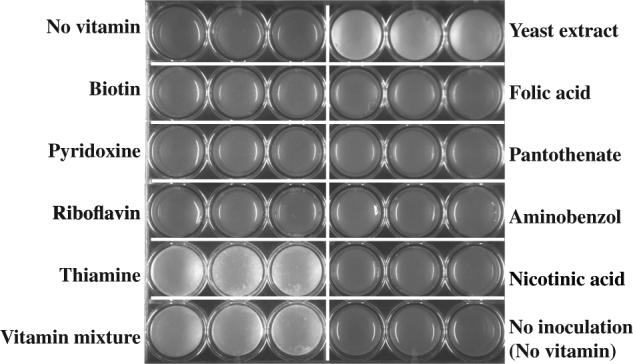
*Serendipita indica* growth under different conditions. Growth on agar plates with defined medium supplemented with different vitamins as shown on each row and column. Each treatment has three replicates presented in three adjacent wells. *Serendipita indica* grows in white colonies. Images were taken after 2 weeks of growth

*Serendipita indica* is an endophytic fungus that can colonize roots of a wide range of plants and can confer a range of beneficial effects, including enhanced plant growth, resistance to biotic and abiotic stresses [34–36], promotion of adventitious root formation in cuttings [37], and assisting phosphate assimilation [38]. Despite its broad host range, *S. indica* also has the ability to grow in the absence of host plants [39]. Exploiting this ability, we attempted to create a fully defined growth medium that was based on previous physiological studies on *S. indica* [39–43]. Using our defined medium, we tested the effect of the vitamins on growth by cultivating *S. indica* in a series of vitamin-free media each supplemented by a specific vitamin (Fig. 1). The results showed that *S. indica* is auxotrophic for thiamine; while none of the other individual vitamin additions supported growth, thiamine and full vitamin addition did. This finding was further confirmed by growing *S. indica* on plates supplemented with an additional agar block only containing a defined amount of thiamine. In this case, growth of *S. indica* resulted in expansion towards the thiamine agar block, suggesting that growth occurs on a thiamine gradient or is linked with an active chemotaxis towards the thiamine source (Supplementary Information; Figure S1). We also quantified the growth of *S. indica* with different concentrations of thiamine and found that hyphae growth and spore formation showed a positive, but saturating, dependency on thiamine levels (Supplementary Information; Figure S2). Without thiamine in the media, we still observed germination and very little hyphal growth (Supplementary Information; Figure S2), possibly supported by spore-stored thiamine. Besides these measurements, we also observed thiamine effects on *S. indica* growth using time-lapse microscopy (see discussion below and videos provided as Supplementary Files 1 and 2).

### *Serendipita indica* auxotrophy is reflected in its genomic enzyme content

**Fig. 2:**
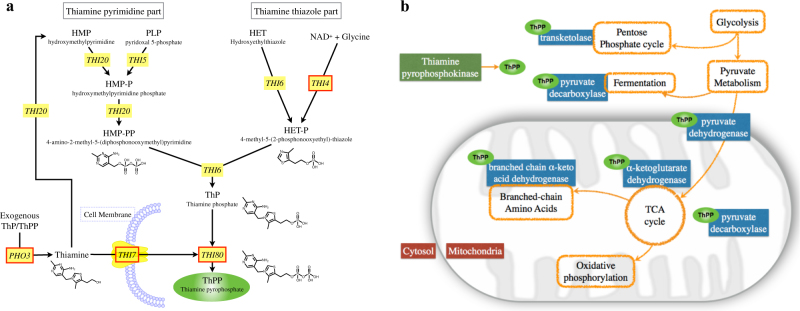
Thiamine-related genes and reactions. **a** Overview of the thiamine biosynthesis pathway in *Saccharomyces cerevisiae* based on Ref. [47], and as included in the KEGG metabolic pathways (Pathway: sce00730) [85]. Yellow boxes indicate genes encoding for the enzymes in the corresponding reactions. Red borders indicate genes for which there are *S. cerevisiae* homologs in *S. indica* (see Methods). **b** Simplified schematic of central metabolism in eukaryotic cells with cytosol and mitochondria compartments indicated. Orange enclosures show reactions of the central metabolism, while blue boxes show essential enzymes catalyzing these reactions and requiring ThPP as a co-factor (shown in green). Green oval indicates ThPP . Red boxes indicate the positions inside a cell

To support and better understand these physiological results, we analyzed the *S. indica* genome for the presence of genes associated with thiamine use, biosynthesis, and transport (Fig. 2a). This bioinformatics analysis revealed that *S. indica* lacks most of the genes of the thiamine biosynthesis pathway (Supporting Table S1 and Fig. 2a). In particular, we did not find any homologs of the genes *THI5, THI6*, and *THI20*. *THI5* encodes the enzyme involved in the synthesis of the thiamine-precursor hydroxymethylpyrimidine (HMP), *THI6* encodes the bifunctional enzyme acting as thiamine phosphate pyrophosphorylase and hydroxyethylthiazole kinase, and *THI20* encodes an HMP kinase that displays both kinase and thiaminase II activity [44]. For the gene *THI4*, which encodes a bifunctional protein involved in thiamine thiazole synthase and mitochondrial DNA damage tolerance [45–47], we found a truncated homolog (11% length coverage against *THI4* sequence in *S. cerevisiae*). Considering all other key biosynthesis genes (*THI5*, *THI6*, and *THI20*) are absent, it is possible that this truncated form relates to mitochondrial DNA damage tolerance [45–47]. We also found that *S. indica* contains a homolog of the *THI7* (or alternative name *THI10*) that encodes a thiamine transporter, and a homolog of the *PHO3* gene, whose product catalyzes dephosphorylation of thiamine phosphate to thiamine, thereby increasing its uptake [45]. The *S. indica* genome also contains *THI80*, which encodes a thiamine pyrophosphokinase catalyzing the conversion of thiamine into thiamine pyrophosphate (ThPP), the active form of thiamine as a key co-factor involved in central metabolic reactions [48]. In particular, ThPP is involved in pyruvate fermentation and conversion for entry into the citric acid cycle (TCA), α-ketoglutarate to succinyl-CoA conversion in the TCA cycle (Fig. 2b and S3), transketolase reactions in the pentose phosphate pathway, and biosynthesis reactions for leucine, isoleucine, and valine [49].

**Fig. 3:**
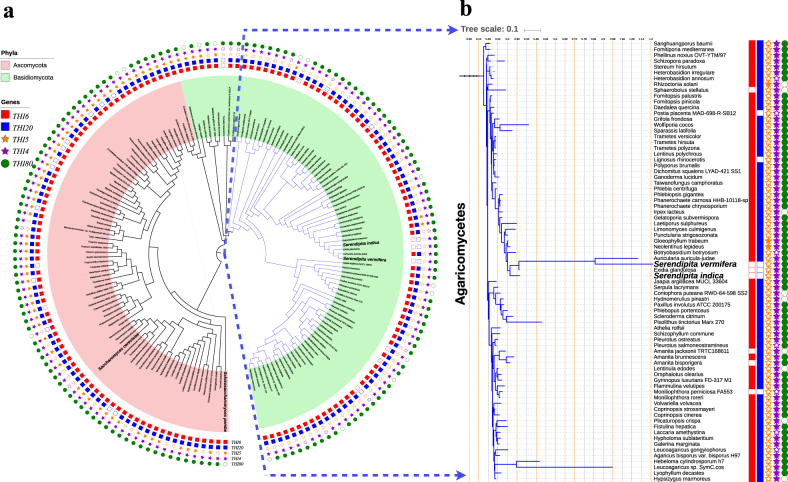
Summary of phylogenetic and bioinformatics analyses.** a** The phylogenetic tree of all 162 fungal species used (see Methods). Key species, *S. cerevisiae*, *S. pombe*, *S. indica*, and *S. vermifera*, are highlighted in bold. The tree contains 77 species from the phylum of *Ascomycota* (red background) and 85 species from the phylum of *Basidiomycota* (green background). Of the latter, 76 species are in the class of *Agaricomycetes* (blue branches). **b** Closer view of the phylogenetic relations among all 76 species in the class of *Agaricomycetes*. On both panels **a** and **b**, the presence/absence of each of the five thiamine biosynthesis genes in each species is indicated with different colors and symbols (as shown in the figure). Filled (empty) symbols indicate presence (absence) of a given species

These results support the experimental observation of thiamine auxotrophy in *S. indica*. To see if such a loss of thiamine biosynthesis is widespread in other fungi, including evolutionarily closely and more distant relatives of *S. indica* and unrelated fungal species, we analyzed the absence and presence of all of the above key genes in available fungal genomes in the phyla of *Basidiomycota* (featuring *S. cerevisiae*) and *Ascomycota* (featuring *S. indica*). We mapped this information onto a phylogenetic tree that we created using the 18s ribosomal gene (see Methods). Summarized in Fig. 3, this analysis showed the same genetic pattern (including the truncated version of the *THI4* homolog) for key thiamine biosynthesis genes in *Serendipita vermifera*, the closest relative of *S. indica*. In addition, we found that within the analyzed species from the *Basidiomycota* phylum, there are five species lacking either *THI20* or *THI6*, and five species lacking both genes (Fig. 3), suggesting that they are unable to synthesize thiamine on their own (Fig. 2a). Among the analyzed species from the *Ascomycota* phylum, there are 14 species lacking either *THI20* or *THI6*, while none of them lack both genes (Fig. 3). Taken together, these findings suggest that a complete or partial loss of thiamine biosynthesis genes can be observed in different fungi from different classes, rather than being confined to a specific class.

### *Bacillus subtilis* complements *S. indica’s* auxotrophy for thiamine and promotes its growth

**Fig. 4:**
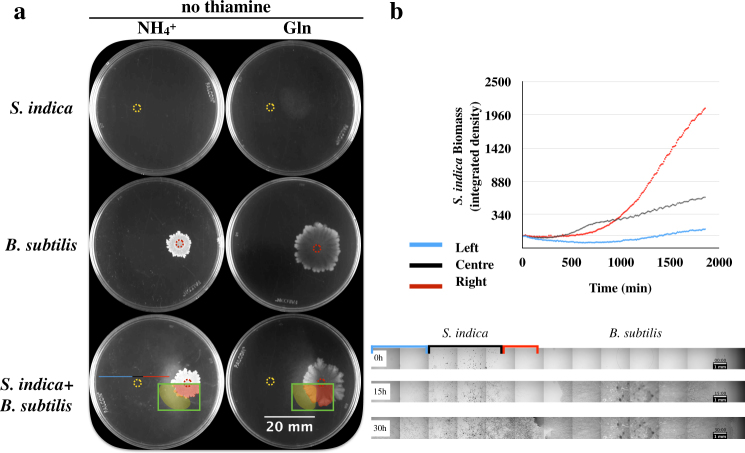
*Serendipita indica* and *B. subtilis* interactions on agar plates. **a** Rows from top to bottom show growth of monocultures of *S. indica*, *B. subtilis*, and their co-culture, respectively, under the absence of thiamine. The yellow dotted circle on the images indicates the *S. indica* inoculation point. Red dotted circle indicates *B. subtilis* inoculation point. The left and right columns show growth on plates after 2 weeks, using ammonium and glutamine as nitrogen sources, respectively. Green square highlights the colonies with pseudo-color, where the area covered by *S. indica* hyphae is shown in yellow, and the *B. subtilis* colony is shown in red. The areas were manually drawn. When both organisms were cultured together (bottom row), *B. subtilis* and *S. indica* were inoculated on the right and left of the plate, respectively. Plates shown are representative of at least three replicates for each condition. We performed two biological replicates of this experiment, with qualitatively similar results. **b**, Top: *S. indica* biomass obtained from the defined horizontal sections of the plate of *S. indica* and *B. subtilis* co-culture on thiamine-free medium (the bottom left plate shown in the third row of part **a**). Biomass was calculated by integrated density of each horizontal sections based on image analysis. The blue, black, and red lines correspond to the horizontal sections as shown on the plate and in the time-lapse image series of *S. indica* and *B. subtilis* growth on agar plates as below. Bottom: The microscopy images are from the middle cross-section of the plate through the inoculation point of *S. indica* and *B. subtilis*, at 0, 15, and 30 h after inoculation. The red, black, and blue highlighted sections correspond to the *S. indica* colony side closer to *B. subtilis*, the middle of the colony, and the colony side far from *B. subtilis*, respectively

Given the crucial role of thiamine-derived co-factors in central metabolism (Fig. 2b), *S. indica* growth in nature apparently depends on environment-derived thiamine. Indeed, thiamine can be synthesized by various bacteria, fungi, and plants [50–52]. Among these, *B. subtilis*, a bacterium commonly found in the soil [53] is an established model organism [54]. *Bacillus subtilis* also requires thiamine for its growth and its thiamine biosynthesis pathways are well studied [50, 55]. Combined with the fact that *B. subtilis* is normally a plant-beneficial microbe [56], this motivated us to explore the possibility that the identified *S. indica* auxotrophy for thiamine could be satisfied upon co-culturing with *B. subtilis*. We created co-cultures of these two species on agar plates using our defined medium and two common nitrogen sources to evaluate possible auxotrophic interactions under these conditions. We found that *B. subtilis* could indeed stimulate *S. indica* growth under thiamine-free conditions and that *S. indica* growth followed a spatial pattern, with significant growth in the vicinity of the *B. subtilis* colony (Fig. 4a). A similar spatial growth was observed in experiments with *S. indica* and a supplied thiamine agar block (Supplementary Information; Figure S1).

We used time-lapse microscopy to quantify this spatial growth pattern of *S. indica* and found that growth (as approximated by image density) happened faster at the side closer to the *B. subtilis* colony compared to the far side of the plate (see Fig. 4b and videos provided as Supplementary Files 3 and 4). This could be explained by the presence of an increasing thiamine gradient towards the *B. subtilis* colony that facilitates *S. indica* hyphal growth. While these findings strongly suggest a *B. subtilis*-linked thiamine provision, which then promotes *S. indica* growth, our attempts to quantify thiamine from agar plate co-cultures has failed, presumably due to a combination of thiamine consumption and sensitivity limitations of available thiamine assays (50 μg/l) [57]. We were, however, able to detect thiamine from concentrated *B. subtilis* cultures, and found the concentration in liquid culture supernatants to be approximately 7.56 μg/l.

While the above findings strongly suggest that the growth enabling of *S. indica* by *B. subtilis* is due to thiamine supply, it is theoretically possible that *B. subtilis* provides metabolites other than thiamine, which allow bypassing of central reactions requiring ThPP as a co-factor. In other words, provision of metabolites that are downstream of pyruvate in the TCA cycle (Fig. 2b). To examine this possibility, we analyzed growth of *S. indica* in the absence of thiamine but supplemented with organic and amino acids that link to the central carbon metabolism. We found that none of the 17 amino acids or 8 organic acids tested or their combinations allowed for *S. indica* growth in the absence of thiamine (Supplementary Information; Figure S4). This finding further confirmed that *B. subtilis* facilitated growth of *S. indica* in thiamine-free medium is linked directly to thiamine.

### Metabolic profiling shows additional metabolic interactions between *S. indica* and *B. subtilis*

**Fig. 5:**
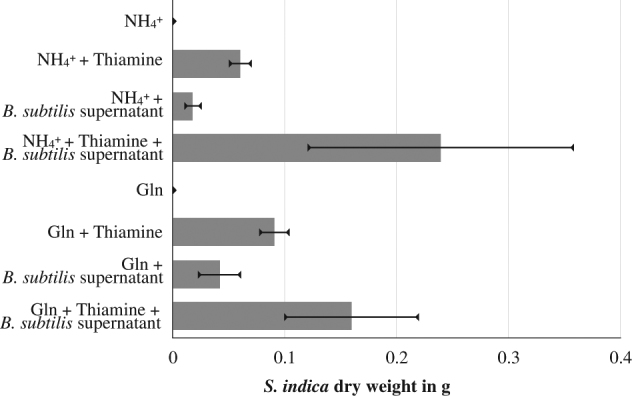
*Serendipita indica* growth under different media compositions. Growth using either ammonium or glutamine and supplemented with thiamine or *B. subtilis* supernatant, as indicated on the *y*-axis. The *x*-axis shows *S. indica* growth approximated by total dry weight after 1 week of growth. Three technical replications were used for generating the weight data. Data shown here is from one of the two biological replications, with others giving quantitatively similar results

To analyze the basis of metabolic interactions between the two organisms and to collect more evidence for thiamine-based auxotrophy, we grew each organism in liquid culture on its own and then cross-cultured the other organism on the supernatant of the first one. As with agar plates, we found that in the absence of thiamine, the *S. indica* growth was limited to spore germination (Fig. 5). When supplemented with *B. subtilis* supernatant, however, *S. indica* showed significantly increased growth in liquid culture (Fig. 5). There was also a growth enhancement of *S. indica* by the *B. subtilis* supernatant when cultured in the presence of thiamine.

To further elucidate these physiological observations, we repeated these experiments and undertook an ion chromatography (IC)-based targeted metabolite quantification (see Methods). In particular, we were able to quantify the key nitrogen compounds (ammonium, glutamine, and glutamic acid), and organic and amino acids relating to the TCA cycle (alanine, glycine, lactate, acetate, pyruvate, and formate) (see Fig. 2 and S3) in the supernatant of each organism before and after cross-cultivation. We found that the supernatant from *B. subtilis* monoculture contained significantly higher amounts of acetate and some formate and pyruvate, and that the extracellular levels for these compounds did not change in the presence or absence of thiamine in the media (Supplementary Information; Figure S5). When *S. indica* was grown in the *B. subtilis* supernatant and in the absence of thiamine, the fungus consumed both acetate and formate and produced pyruvate (Supplementary Information; Figure S5). In the presence of both thiamine and the *B. subtilis* supernatant, the consumption of acetate and formate was also observed, but there was also production of lactate in addition to pyruvate (Supplementary Information; Figure S5). There were also few peaks in the IC chromatograms that are altered during the cross-supernatant experiment and that could not be identified (Supplementary Information; Figure S6). In the case of the key nitrogen compounds and amino acids, we found that in glutamine media, *B. subtilis* supernatant contained glutamic acid and glutamine, which were subsequently consumed by *S. indica* (Supplementary Information; Figure S7). In ammonium-based media, we found significant amounts of ammonia unconsumed in the *B. subtilis* supernatant, which is then subsequently consumed by *S. indica*. This ammonia consumption by *S. indica* led to the accumulation of glutamic acid, glutamine, alanine, and small amounts of glycine (Supplementary Information; Figure S7).

These findings, in particular acetate and formate cross-feeding from *B. subtilis* to *S. indica*, explain the positive impact of *B. subtilis* supernatant on growth irrespective of thiamine availability. They also provide further support that the *B. subtilis-*associated growth of *S. indica* relates to thiamine provision rather than organic acids, since acetate and formate alone did not enable *S. indica* growth in thiamine-free media (Supplementary Information; Figure S4).

### The successful co-existence of *S. indica* and *B. subtilis* depends on spatiotemporal organization in the system

**Fig. 6:**
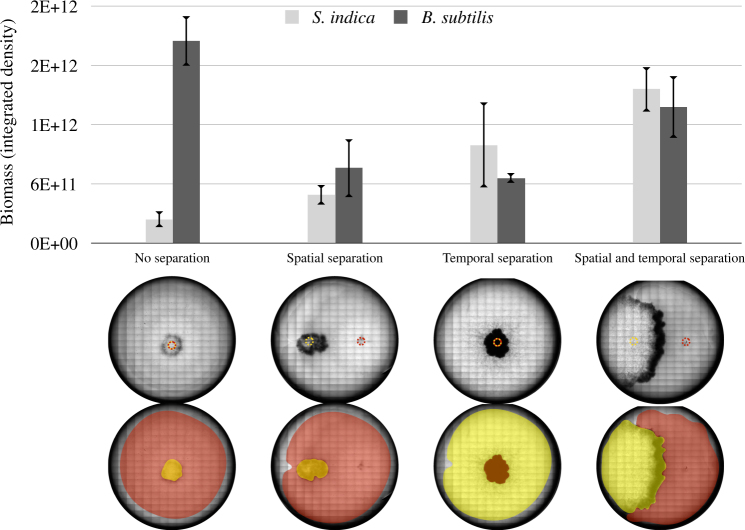
Biomass of *B. subtilis* and *S. indica* under different spatiotemporal culturing cases. “No separation” refers to *B. subtilis* culture and *S. indica *spores being pre-mixed at 1:1 volume ratio, and then inoculated as a single solution. “Spatial separation” refers to approximately 1.5 cm separation of *S. indica* (left) and *B. subtilis* (right) inoculation points. “Temporal separation” refers to inoculation of *S. indica* 3 days prior to *B. subtilis* inoculation. The yellow dotted circle on the images indicates the *S. indica* inoculation point. Red dotted circle indicates *B. subtilis* inoculation point. Below the graph are shown the plate images. The pseudo-coloring indicates *S. indica* hyphal area (in yellow) and *B. subtilis* colony (in red). The area of each colonies was manually drawn. Growth of the different species was approximated by tracing their respective colonies on the plate and measuring the image intensity from the engulfing areas 2 weeks after *S. indica* inoculation. Measurements are from three replicate agar plates, with a representative plate image shown at the bottom. These images show microscopic scans of each plate at 2 weeks of growth. For the “no separation” case, there was no observable *S. indica* colony expansion after 1 week. We performed four biological repetitions of this experiment, with qualitatively similar results

The above findings show that *B. subtilis* can support the growth of *S. indica* in thiamine-free medium either through its supernatant or when co-cultured at a distance on an agar plate. Both experimental setups were geared towards identifying possible interactions among the two species through utilization of the excretions of one species by the other, but did not necessarily consider the spatiotemporal factors on such interactions. Thus, the remaining question was whether both species could still co-exist and establish a successful interaction under different conditions regarding the spatial proximity or size of initial inoculation, or the actual growth phase that the different species are in at the time of introduction onto the agar. While addressing these questions is experimentally challenging, we attempted here to analyze the impact of spatial-temporal factors on the outcome of the *S. indica*–*B. subtilis* auxotrophic interaction by changing inoculation time and location on agar plates. In particular, we separated the inoculation of *S. indica* spores from *B. subtilis* inoculation either in time (by inoculating spores 3 days before *B. subtilis* colony inoculation) or space (by inoculating *S. indica* and *B. subtilis* at certain distance to each other) (see Methods). Alternatively, we inoculated *S. indica* spores after mixing with *B. subtilis*. These experiments mimic a scenario commonly found in agrotechnology practices when using pre-mixed cultures or spores of different microbes as soil biofertilizers or plant seed pre-treatments [58]. We found that with temporal or spatial separation, both species could successfully grow in the absence of thiamine, indicating a positive auxotrophic interaction (Fig. 6). In contrast, direct co-inoculation of *B. subtilis* with *S. indica* significantly hampered co-existence of the two species, particularly reducing *S. indica* growth (Fig. 6).

One possible explanation for this observed strong effect of co-inoculation is that alterations of the micro-environment by one species causes inhibitory effects on the other. Indeed, previous studies have shown that microbial growth on agar plates can create oxygen depletion within a colony [24, 25, 59]. This explanation could be particularly relevant in our experiments, as germination and initial growth of some soil fungi are shown to require substantial oxygen [60]. To gain insight on oxygen levels in our experimental system, we have repeated the experiments shown in Fig. 6, and measured oxygen levels on the agar plate (see Methods). We could confirm, as observed before [59], that oxygen is completely depleted on the *B. subtilis* colonies, already within few hundred microns underneath the surface of the plate/colony (Figure S8). In contrast, oxygen levels on the inoculation and growth zone of *S. indica*, as well as elsewhere on the agar plate remained high to significant depths from agar/colony surface (Supplementary Information; Figure S8). These results support the hypothesis that oxygen depletion by *B. subtilis* might inhibit germination of *S. indica* spores in “pre-mixed” inoculations.

## Discussion and conclusions

Here, we report an auxotrophy for thiamine in the endophytic fungus *S. indica* and how this thiamine requirement can be satisfied by *B. subtilis*. We show that *S. indica* growth in thiamine-free medium cannot be supported by any other vitamin or relevant organic and amino acid, but can be restored when co-cultured with *B. subtilis* or with its liquid culture supernatant. While these co-culturing experiments identified additional metabolic interactions between these two species, they support a primary and successful auxotrophic interaction between the two organisms.

The presented experimental findings on thiamine auxotrophy are supported by bioinformatics analyses, showing that *S. indica* and its close relative *S. vermifera* lack the key thiamine biosynthetic genes *THI5*, *THI6*, and *THI20*, and only contain a truncated version of the *THI4* gene. Instead, both organisms possess gene homologs encoding for proteins involved in the efficient transport of thiamine and its conversion into an active form (*THI7*, *PHO3*, and *THI80*, respectively). A phylogenetic analysis shows that these genetic patterns are not unique to these two species, but also not widespread, indicating that loss of thiamine biosynthesis can be related to their ecology and life style (both species are general plant colonizers [31]. Within an environment where thiamine is readily available, it is indeed possible that thiamine biosynthesis is lost due to enzymatic costs. In particular, the protein product of *THI4* is shown to undergo only a single turnover before requiring re-synthesis, a so-called “suicide enzyme” [61]. Due to this potential high cost, loss of *THI4-*related thiamine biosynthesis might be adaptive under circumstances where thiamine can be acquired from the environment [48]. In both *S. indica* and its close relative *S. vermifera* genome there is only a truncated *THI4* gene (11% coverage against *THI4* sequence in *S. cerevisiae*), and it is possible that this gene relates only to the known additional function in mitochondrial DNA repair [46, 61] rather than thiamine biosynthesis (as the other thiamine biosynthesis *THI5*, *THI6*, and *THI20* are absent).

Interestingly, we found that the auxotrophic interaction between *S. indica* and *B. subtilis* can only be achieved under conditions where the inoculation (and germination) of the two species is separated in time or space. This finding adds to an array of recent studies demonstrating the importance of spatiotemporal organization for the emergence and maintenances of metabolic interactions in microbial and other systems [15, 20–23]. In general, such effects can emerge from a multitude of factors including competition for local resources, spatial factors such as mixing and physical contact, or changes in ecological conditions caused by species' activities. While a detailed elimination of each possible factor might be difficult in specific cases, our findings suggest that in the case of *S. indica* and *B. subtilis*, oxygen depletion by a growing *B. subtilis* colony can cause inhibition of *S. indica* seed germination, and result in the failure of any auxotrophic interaction from establishing. Observations on oxygen depletion in growing bacterial colonies has been made before [24, 25, 59], as well as the idea that changing micro-environment conditions can influence the establishment and maintenance of microbial interactions [10, 15, 23, 62, 63].

Emerging studies have revealed the extent and importance of metabolic interactions within co-cultures and larger microbial communities using metabolomics approaches [9, 10, 63]. In the presented study, we focused on characterizing the co-culture dynamics given the primary auxotrophic interaction via thiamine. We have also used a targeted metabolomics approach to identify additional metabolic interactions, involving key organic and amino acids related to primary metabolism (in particular the TCA cycle). These additional interactions hint to the role of overflow fluxes from central metabolism in creating new or additional metabolic interactions that can operate on top of auxotrophic interactions. It is also possible that these different types of interactions can facilitate each other’s emergence and stabilization. For example, in the case of thiamine auxotrophy, limitations in thiamine levels can decrease the rates of pyruvate and α-ketoglutarate processing through the TCA cycle, leading to diversion into amino acids such as glutamic acid, glutamine, alanine, and glycine (see Supplementary Information; Figure S3), which can then create new metabolic interactions. Thus, overflow metabolism and its relation to the availability of electron acceptors or co-factors could provide a conceptual framework to understand and study microbial interactions [64, 65]. This view is also supported by recent studies indicating the role of both carbon and nitrogen overflows in the establishment of metabolic interactions [23, 63, 66].

The presented findings have implications both for the study of *S. indica*, as an important plant-supporting soil fungus [41], and for the engineering and application of minimal synthetic communities that aim to establish plant-supporting soil communities. In the former direction, future metabolic and physiological studies of *S. indica* will be enabled by the defined media conditions and identified thiamine auxotrophy in this study. A key suggestion from a biotechnological perspective, for example, is to consider thiamine as an important factor in the commercial mass production of *S. indica* [67]. In the latter direction, the finding that the success of auxotrophic interactions relates to spatiotemporal effects suggests that consideration should be given to inoculation timing when designing or applying biofertilizers or bio-control agents to the soil. Indeed, microbial interactions and synergisms are suggested to be crucial for soil fertility, bioproductivity, and ecosystem functioning [68–70]. Plants significantly benefit from symbioses with soil microbes, with benefits ranging from nutrient supply, growth promotion to elevating plant stress resistance [56, 71–74]. At the same time, soil microbes can interact among themselves or alter each other’s interactions with the plants [75–78]. The biochemical basis of these potential multi-level interactions in the soil has remained mostly elusive to date, with few documented cases of amino acid auxotrophies in specific soil bacteria and vitamin provision from plants relating to their root colonization [79–81]. The presented synthetic community of *S. indica* and *B. subtilis* shows that metabolic auxotrophy can directly underpin microbial interactions and growth, and that the success of interactions can be determined by spatiotemporal organization in the system. This synthetic system allows controlled investigations (and potential optimization) of fungal–bacteria interactions and can be further extended with additional microbes and a plant. The resulting minimalist synthetic ecosystem can provide a platform to analyze and control cross-kingdom relationships between plants and their growth-promoting fungi and bacteria [90], and enable new applications for vertical farming and crop production in the future.

## Materials and methods

### *Serendipita indica* cultures, growth media, and conditions

The defined basic medium for testing *S. indica* growth with ammonium as nitrogen source contained per liter; 15 g agar, 20 g glucose, 1.32 g (NH_4_)_2_SO_4_, 0.89 g Na_2_HPO_4_·2H_2_O, 0.68 g KH_2_PO_4_, 35 mg Na_2_MoO_7_·2H_2_O, 5.2 mg MgSO_4_, 2.5 mg FeSO_4_·7H_2_O, 0.74 mg CaCl_2_·2H_2_O, 0.0043 mg ZnSO_4_·7H_2_O, and 0.004 mg CuSO_4_·5H_2_O. Growth experiments for testing effects of different vitamins were performed in 24-well plates (Ref: 353226, Falcon), where each well contained 2 ml of the basic medium, supplemented with either 200 μg/l of single vitamin solution, 1 g/l yeast extract, 200 μg/l mixture of all eight vitamins tested, or equivalent amount of deionized water. Each well was then inoculated with 1 μl of *S. indica* spore suspension (approximately 500,000 spores/ml, counted with Neubauer counting chamber), where spores were harvested from 6 to 8 weeks old *S. indica* agar plates. In the “non-inoculated” control treatment, 1 μl of deionized water was used instead. Each treatment condition was prepared in three technical replicates. The 24-well plates were then sealed with parafilm and placed in a 30°C static incubator for 2 weeks. Images were taken with a gel doc system (Syngene) at the end of this period.

### Experiments on agar plates, growth media, and conditions

The defined (basic) medium for testing *S. indica* growth on agar plates is given as above. When the chosen nitrogen source was ammonium, 1.32 g/l (NH_4_)_2_SO_4_ was added to this basic recipe. When the chosen nitrogen source was glutamine, 1.46 g/l glutamine was added to this basic recipe. For experiments with thiamine, 150 μg/l thiamine was added to the basic media after autoclaving.

Experiments were carried out on 60 mm dishes, filled with 6 ml of agar medium given above. A 500,000 spores/ml *S. indica* spore suspension, with spores harvested from 6 to 8 weeks old *S. indica* agar plates, was inoculated with 2 μl (on the left side of the plates). At approximately 2 cm distance to the right of the inoculum, either a “mock” solution, a thiamine block or a *B. subtilis* inoculum were placed. The “mock” solution was 2 μl of sterile water. The thiamine blocks were made by pouring 6 ml of 1.5% agar solution containing150 μg/l thiamine into a 60 mm plate, and then punching a block out using a sterile pipette tip with a diameter of 4.7 mm. Therefore, each agar block used contained approximately 5.6 ng thiamine. The *B. subtilis* inoculum was a 2 μl sample harvested from a culture, grown in 5 ml liquid Lysogeny broth (LB) [83] to an OD_600_ ≈ 0.5 measured by spectrophotometer (Spectronic 200, Thermo Fisher Scientific), then washed and re-suspend to OD_600_ ≈ 0.5 with 10 mM MgCl_2_. The plates were incubated in 30°C for 2 weeks. Images were taken with gel doc system (Syngene) at the end of this period.

### Time-lapse microscopy and image analysis

Time-lapse microscopy was performed on agar medium cultures that were prepared using the same basic medium described above. A 6-well tissue culture plate (Ref: 353046, Falcon) was used and 1 μl of *S. indica*, *B. subtilis* or mock solution prepared as described above were inoculated on each well accordingly to experiment design. An Olympus IX83 microscope, UPlanFLN ×4 objective and cellSens software were used for recording the growth. Okolab stage top incubator (H301-T-UNIT-BL-Plus system, and H301-EC chamber) were used for incubation, with a temperature sensor and lens heater set to 30°C and stabilized for at least 2 h prior to the experiment. Different fields of view were chosen at interior and periphery of each colony and images from those fields were recorded using the automated microscope stage and Olympus cellSens software. Images were taken in 1 h intervals and put together as image series. ImageJ (Fiji) [84] was used for measuring the mean intensity on each field of view over time, normalized against the intensity value of the first frame from each view point (as shown in Fig. 4).

### Spatial and temporal separation experiments

*Serendipita indica* (500,000 spores/ml, determined by counting with Neubauer counting chamber) and *B. subtilis* (OD_600 _≈ 0.5, determined by spectrophotometer (Spectronic 200, Thermo Fisher Scientific)) were cultivated on thiamine-free synthetic medium containing ammonium as sole nitrogen source, and using six-well tissue culture plates (Ref: 353046, Falcon). On each plate, 1 μl of *S. indica* and 1 μl of *B. subtilis* were inoculated on 5 of the wells; one well was intentionally left non-inoculated as a blank. In the “no separation” case, *S. indica* and *B. subtilis* were pre-mixed at 1:1 volume ration, and inoculated on the center of each well. For “spatial separation” case, *S. indica* was inoculated 7.5 mm left to the center of a well and *B. subtilis* 7.5 mm right to the center, leaving 15 mm distance in between. For “temporal separation” case, *S. indica* was inoculated on each well, the plates were then incubated for 3 days and *B. subtilis* was inoculated after this time. The 3-day time separation was primarily based on the germination time of *S. indica* spores, which can take 2–3 days in our synthetic medium. As shown in the video included as Supplementary File S1, *S. indica* is able to germinate and grow a low amount of hyphae, potentially with available thiamine stored in spores. All the plates were incubated in 30°C for 2 weeks (starting from the time of *S. indica* inoculation). Images were taken by scanning each well under a microscope (Olympus IX83) using the same exposure time under bright field. ImageJ was used for measuring the biomass by integration of the total colony density. An image of each colony was manually outlined using the selection tool. The selected area was compared with the same location on a blank well from the same plate. The area and relative intensity were recorded (using “measure” function) and used for calculating the colony growth.

### Oxygen measurements

Oxygen measurements were done in a replicate experiment with the “spatial separation” and “no separation” treatments described above and using with thiamine-free agar medium containing glutamine as sole nitrogen source. UniSense micro-electrode (OX-NP with Unisense microsensor multimeter and Unisense Sensor Trace Suite software) was used for daily oxygen measurements. Three identical plates of the same treatment and initiated on the same day was used for each measurement (i.e., three plates per day per treatment) and discarded afterwards. On each day, the micro-electrode was calibrated using aerated distilled water (air bubbled through for 3–5 min) and an anaerobic solution (prepared as per the manufacturer's instructions; 0.1 M ascorbic acid in 0.2 M NaOH, mixed briefly to dissolve and left unagitated for calibration 5 min prior to first measurement). For each measurement, the electrode was first placed on the colony or agar surface. Once a measurement at the surface was done, the electrode was lowered into the agar or colony by means of a manual micro-manipulator (Scientifica), and at 400 μm intervals in the z-dimension of the manipulator. Three depths (0.4, 0.8, and 1.2 mm from the surface) were used for oxygen measurement. Measurements were taken on *B. subtilis*, *S. indica*, and pre-mixed co-culture inoculation points, as well as on an off-location without any bacteria or fungi growth.

### Supernatant cross-feeding experiments and metabolite analysis

Axenic cultures of *S. indica* and *B. subtilis* were cultivated in 50 ml basic medium described above, with or without thiamine. For *S. indica* an inoculum of 50 μl of a 500,000 spores/ml spore suspension, harvested from 6 to 8 weeks old *S. indica* agar plates, was used. For *B. subtilis*, an inoculum of 50 μl, sampled from a culture grown in LB to an OD_600 _≈ 0.5 determined by spectrophotometer (Spectronic 200, Thermo Fisher Scientific), and washed with 10 mM MgCl_2_, was used. After 1 week incubation in 30°C and at 150 rpm, *S. indica* cells were harvested by centrifugation at 18,000 × *g* for 20 min. The supernatant was collected and filtered through a 0.2 μm polyethersulfone (PES) filter (Ref: WHA67802502, Whatman), while biomass was washed with 40 ml MilliQ water, dried using a centrifugal evaporator (EZ-2 Elite, Genevac), and then weighted. The growth of *B. subtilis* liquid cultures was monitored daily by taking 1 ml samples and measuring OD_600_ by spectrophotometer (Spectronic 200, Thermo Fisher Scientific). At the end of 1 week, the remaining liquid culture was centrifuged at 18,000 × *g* for 10 min. The supernatant was collected and filtered through a 0.2  μm PES filter, while biomass was discarded.

Both supernatants were mixed with fresh basic medium in a 1:1 ratio to set up new axenic cultures of *S. indica* and *B. subtilis*. One milliliter of liquid samples were collected from these cultures after 1 week by filtering through a 0.2 μm nylon membrane (Ref: WHA7402004, Whatman). Samples were transferred into polypropylene vials (Ref: 079812, Thermo Fisher Scientific) for IC, which was performed using Dionex ICS-500^+^ and column Dionex IonPac AS11-HC-4 μm (2 × 250 mm).

The same samples were sent for commercial amino acid analysis (performed by Genaxxon, Ulm, Germany). A polymeric cation exchanger was used to separate amino acids by high-performance liquid chromatography chromatography (particle size: 5  μm; column dimensions: 125 × 4.6 mm ID) (Amino Acid Analyzer LC3000). Separated amino acids were detected by post-column Ninhydrin derivatization at 125 °C and photometric measurement at 570 nm.

### Sequence analysis and BLAST search of key thiamine biosynthesis genes

Sequences of key thiamine biosynthesis enzymes (Table S1) from *Saccharomyces cerevisiae* S288c were compared with available *S. indica* genome homologs using Position-Specific Initiated BLAST (https://blast.ncbi.nlm.nih.gov/Blast.cgi), to identify the putative functions of the corresponding genes. An *e*value of 1e−6 was chosen as cut-off to identify homologous sequences [85]. The results of the analysis are shown in Table S1.

### Phylogenetic and bioinformatics analysis

In order to understand the loss of thiamine biosynthesis function in *S. indica*, we performed a phylogenetic analysis of 162 fungi species including *S. indica* and its close relative *S. vermifera*, and focusing on thiamine biosynthesis genes. This focus meant that we can only use species with fully sequenced and well-annotated genomes. To collate fungi species fulfilling these criteria, we first used the KEGG database [86] in which there are 102 species with annotated genomes that are mapped to metabolic pathways. This dataset, however, contains only 16 species in the *Agaricomycetes* class, in which the species *S. indica* and *S. vermifera* belong. To get more close relatives of *S. indica*, we used the NCBI Taxonomy [87] and SILVA rRNA [88] databases and collated 60 species that are from the *Agaricomycetes* class and that have full genome assembly information. For the resulting 162 species, we downloaded SSU-rRNA sequences from SILVA rRNA database [88]. Where multiple sequences were present for a given species, we used the longest sequence with highest quality. We then aligned the resulting 162 rRNA sequences using the MUSCLE tool [89], and built a maximum-likelihood phylogenetic tree using PhyML [90]. Onto the resulting phylogenetic tree, we mapped the presence and absence information of the thiamine biosynthesis genes *THI6*, *THI20*, *THI5*, *THI4*, and *THI80* (see Fig. 3). This information was already available for the 102 species that are contained in the KEGG database. To obtain it for the additional 60 species from the *Agaricomycetes* class (and including the species *S. indica* and *S. vermifera*), we ran a BLAST analysis, in which we queried each of these species genomes against the *THI6*, *THI20*, *THI5*, *THI4*, and *THI80* gene sequences from *S. cerevisiae*. A gene was considered present in a given genome if there was a match of homologous sequences with an *e-*value smaller than 1e−6 [85].

### Thiamine measurements on *B. subtilis* liquid cultures

Axenic cultures of *B. subtilis* were cultivated in 50 ml synthetic medium containing glutamine as a sole nitrogen source without thiamine. *Bacillus subtilis* inoculum of 50 μl, sampled from a culture grown in LB to OD_600 _≈ 0.5 determined by spectrophotometer (Spectronic 200, Thermo Fisher Scientific), and washed with 10 mM MgCl_2_ was used. After 1 week of incubation in 30°C and at 150 rpm, *B. subtilis* cultures were harvested by centrifugation at 18,000 × *g* for 10 min. The supernatant was collected and filtered through a 0.2 nm PES membrane filter (Ref: WHA67802502, Whatman). The supernatant of 250 μl from each culture was transferred to a clean 1.5 ml Eppendorf tube, followed by sequentially adding 10 μl 1% K_3_[Fe(CN)_6_], 150 μl 15% NaOH solution, and 150 μl isobutanol. The tubes were shaken vigorously for 1 min, followed by 2 min of centrifugation at 13,000 × *g*. The upper isobutanol layer of each tube was transferred to a new 1.5 ml Eppendorf tube, containing approximately 0.2 g Na_2_SO_4_. The tubes were mixed well and centrifuged for 1 min at 13,000 × *g* for solids to settle. One hundred microliters of supernatant from each tube were transferred to 96-well plates (Ref: 3916, black flat bottom, Corning) and the fluorescence was measured using a plate reader (CLARIOstar, BMG Labtech) at 365 nm excitation and 450 emission. The concentration of thiamine was determined with a series of known concentration standard thiamine solutions under the same treatment.

### *Serendipita indica* growth under different thiamine concentrations

Synthetic medium containing ammonium as sole nitrogen source was used for testing *S. indica* growth in different thiamine concentrations. Media containing final thiamine concentrations of 0, 1.5, 15, 150, and 1500 μg/l were prepared, and distributed in a 6-well tissue culture plate (Ref: 353046, Falcon). Each 6-well plate contained one concentration condition, with 3 ml medium in each well. On each plate, 1 μl of *S. indica* (500,000 spores/ml) were inoculated on the center of five wells, while one well was intentionally left non-inoculated as a blank. Plates were incubated at 30°C for 2 weeks. Afterwards, lids were removed and OD_600_ and fluorescence at 365 nm for excitation and 450 nm for emission were measured using a plate reader (CLARIOstar, BMG Labtech) and the plate scan function to get an overall reading of each well. Images of plates were taken using a gel doc system (Syngene).

## Electronic supplementary material

Supplementary File 5(XLSX 22 kb)

Supplementary Information (containing Supplementary Figures and Tables)(PDF 6688 kb)

Supplementary File 3

Supplementary File 4

Supplementary File 1

Supplementary File 2
