# Supplementary material for: Impact of spatial organization on a novel auxotrophic interaction among soil microbes
Source: ISME J. 2018 Mar 23;12(6):1443–56. doi: 10.1038/s41396-018-0095-z (PMC5955953; doi:10.1038/s41396-018-0095-z)
Supplement: Supplementary file 2 — Supplementary Information (containing Supplementary Figures and Tables)(PDF 6688 kb) [file 41396_2018_95_MOESM2_ESM.pdf]

Impact of spatial organization on a novel auxotrophic interaction among soil microbes.

Xue Jiang[1], Christian Zerfaß[1,2], Song Feng[3], Ruth Eichmann[1], Munehiro Asally[1,2], Patrick Schäfer*[1,2], Orkun S Soyer*[1,2]

Running title: Impact of spatial organization on a novel auxotrophic interaction

Affiliations:

[1] School of Life Sciences, The University of Warwick, Coventry, CV7 4AL, United Kingdom

[2] Warwick Integrative Synthetic Biology Centre, The University of Warwick, Coventry, CV7 4AL, United Kingdom

[3] Center for Nonlinear Studies, Theoretical Division (T-6), Los Alamos National Laboratory, Los Alamos, NM87545, USA.

***For correspondence:** Patrick Schäfer, School of Life Sciences, The University of Warwick, Coventry, CV4 7AL, United Kingdom. Phone: +44(0)2476575244. E-mail: P.Schafer@warwick.ac.uk

Orkun S Soyer, School of Life Sciences, The University of Warwick, Coventry, CV4 7AL, United Kingdom. Phone: +44(0)2476572968. E-mail: O.Soyer@warwick.ac.uk

SUPPLEMENTARY FIGURES, TABLE, AND FILES

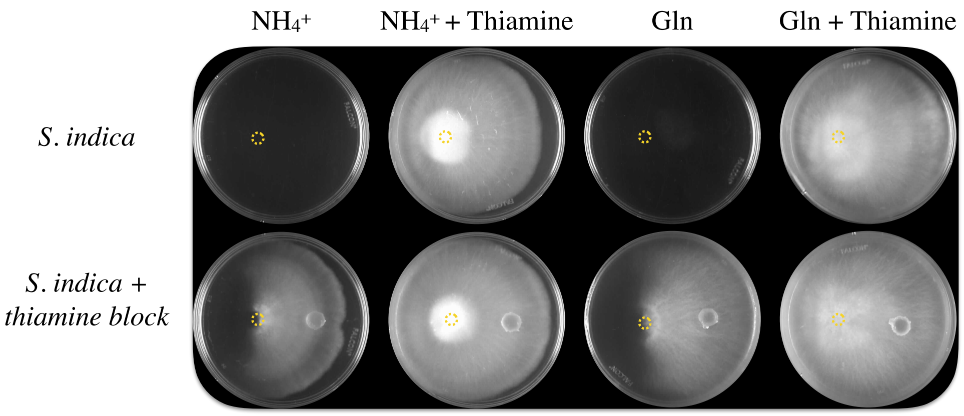

Supplementary Figure 1. *S. indica* growth on agar plates after two weeks. Columns show different media containing thiamine or not and using ammonium and glutamine as nitrogen sources (shown as "NH_4^+" or "Gln" on the panels). The top and bottom rows show the images of agar plates without or with an additional agar block containing thiamine, placed ~1.5 cm to the right side of *S. indica* inoculation point, which is indicated with a yellow circle.

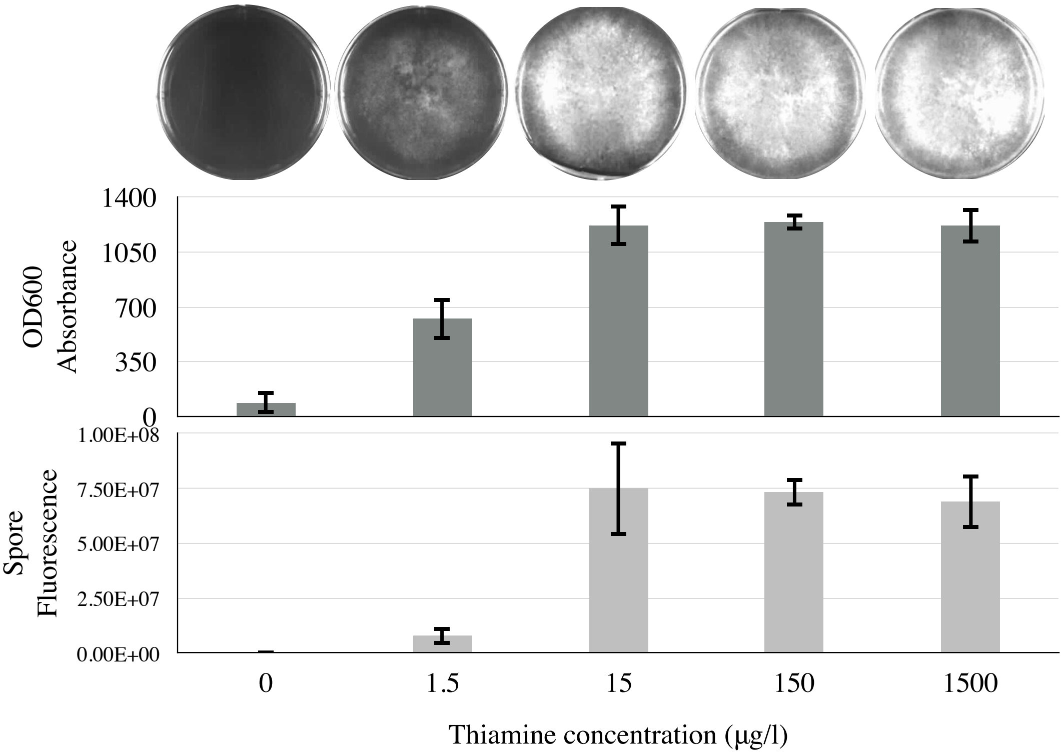

Supplementary Figure 2. *S. indica* growth on medium containing different concentrations of thiamine and ammonium as nitrogen source. Images at top show two weeks growth of *S. indica* on agar plates, and at different concentrations of thiamine as shown below on the bottom *x*-axis. Each condition was repeated 6 times and images shown here are representatives for each condition. Upper and lower bar-plots show plate absorbance at OD_{600} and fluorescence intensity (measured at 390 excitation and 470 emission for detection of *S. indica* spores) respectively.

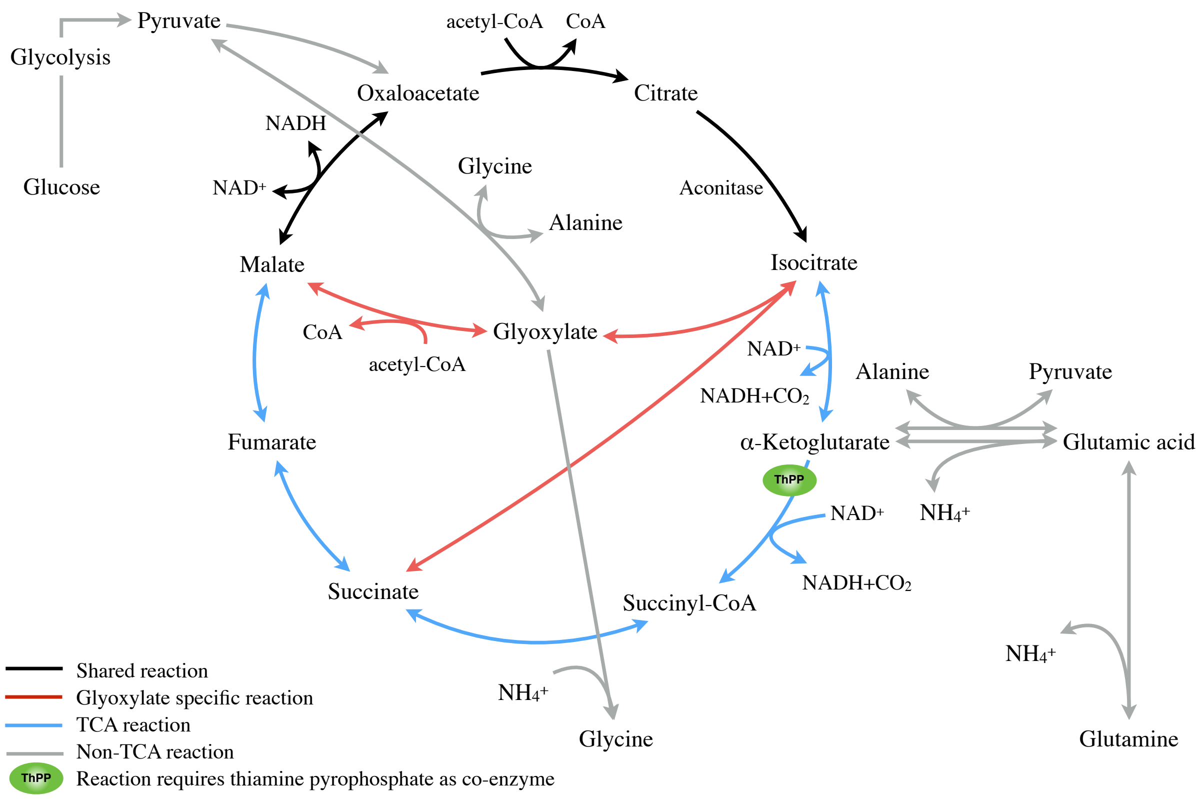

Supplementary Figure 3. Simplified schematic of TCA and glyoxylate cycles from the central metabolism. Black line indicates reactions shared in both glyoxylate cycle and TCA cycle. Red and blue lines indicate reactions specific to the glyoxylate and TCA cycle, respectively. Grey line indicates reactions not presented in either cycle.

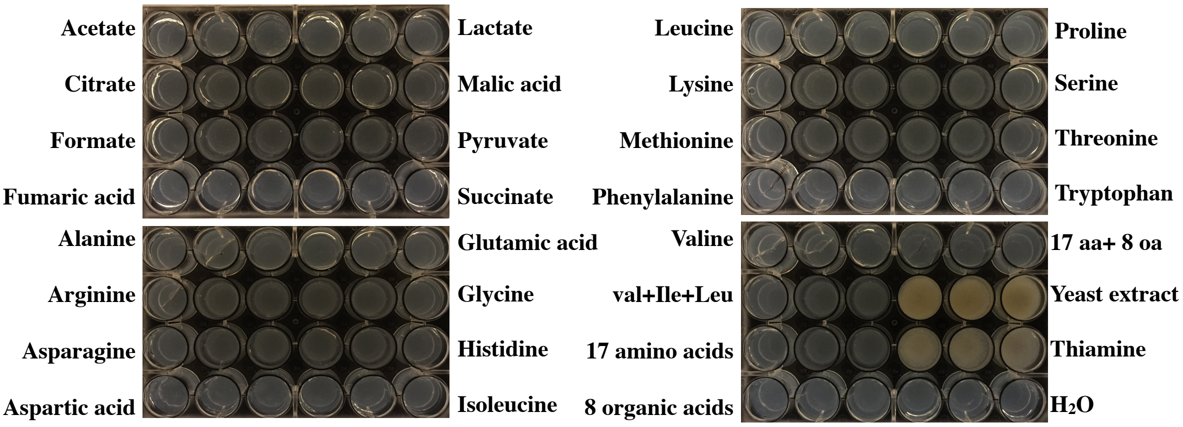

Supplementary Figure 4. *S. indica* amino acid and organic acid screen. *S. indica* growth on the media containing different amino acid or organic acid as supplement. Each treatment has three replicates presented in 3 adjacent wells. *S. indica* growth is visible as white-yellow colonies on the surface of a well. Images were taken after two weeks of growth.

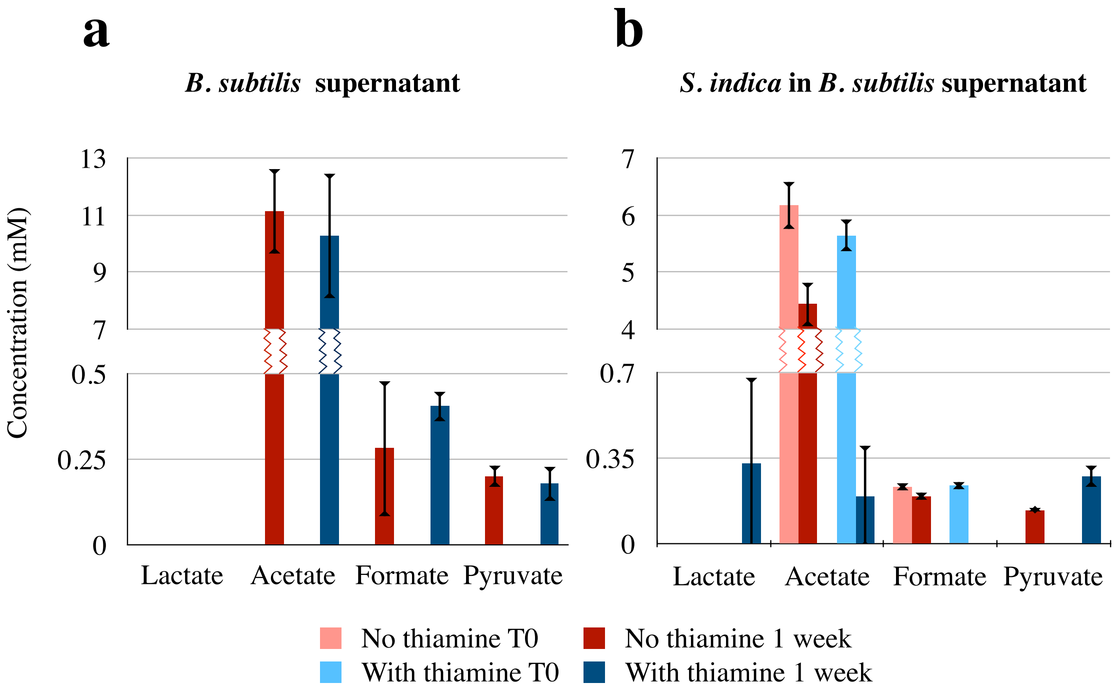

Supplementary Figure 5. Concentrations of key organic acids in the supernatant of *B. subtilis* (**a**), and *S. indica* when grown in *B. subtilis* supernatant (**b**). **a.** The concentration of 4 organic acids accumulated in the supernatant after one week of *B. subtilis* cultivation in liquid medium containing glutamine as sole nitrogen source. **b.** The concentration of 4 organic acids in the supernatant after one week of cultivation of *S. indica* in *B. subtilis* supernatant (**a**) mixed with the same volume of fresh medium. T0 and 1 week indicate the initial condition and after 1 week of growth respectively, while the different colors indicate the thiamine presence/absence as shown in the legend. Note that T0 concentrations in **b** chart correspond to concentrations in **a** chart diluted with same volume fresh media.

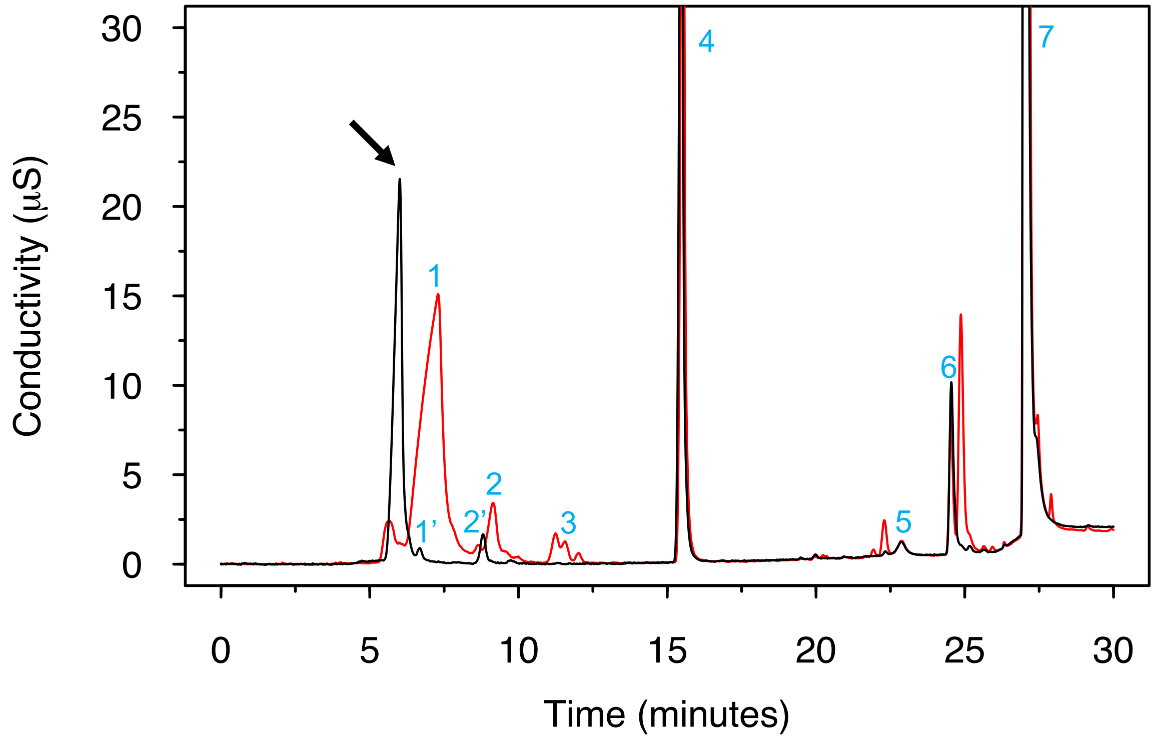

Supplementary Figure 6. Sample chromatogram from IC analysis of cross-supernatant experiments between *S. indica* and *B. subtilis*. The black and red traces are for *S. indica* supernatant (diluted with an equal volume of fresh medium), and *B. subtilis* supernatant after growth in this *S. indica* supernatant, respectively. Several IC-peaks, labelled with blue numbers (with corresponding compound names given below), were identified based on retention times of known reference samples, while few other peaks remained un-identified. One prominent peak (black arrow) derived from *S. indica* culture and disappeared upon culturing *B. subtilis*. Note that some retention times are shifted because of high sample load (i.e. acetate labelled 1/1'), which also affected adjacent peaks (i.e. formate, 2/2'). Chromatograms shown are representative for a triplicate analysis. The identified peaks derive from (1) acetate, (2) formate, (3) pyruvate, (4) chloride, (5) carbonate, (6) sulfate, (7) phosphate.

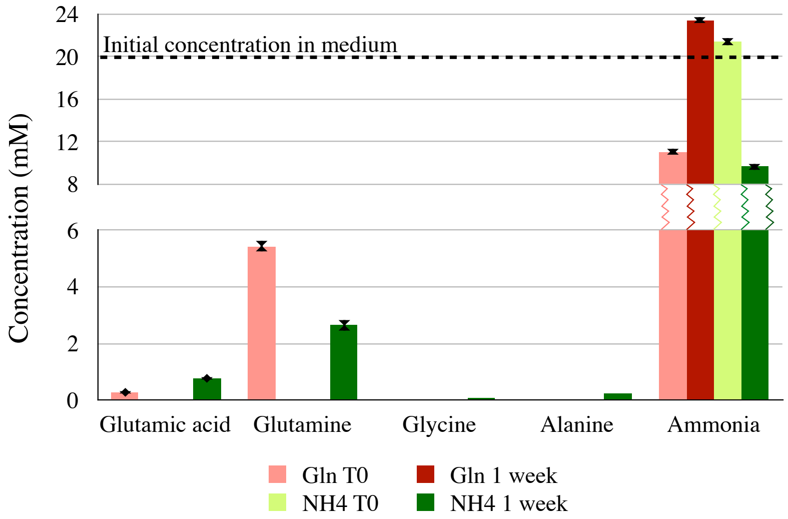

Supplementary Figure 7. Concentrations of detected amino acids and ammonia in the supernatant of *B. subtilis*, and in the supernatant after subsequently cultivation of *S. indica*. The labels "Gln" and "NH4" indicate the use of glutamine or ammonium as sole nitrogen source. "Gln T0" and "NH4 T0" indicate the initial conditions, which is the mixture of supernatant from one-week *B. subtilis* culture and equal volume of fresh medium. "Gln 1 week" and "NH4 1 week" indicate the supernatant after one-week cultivation of *S. indica* in the initial condition. Black dotted line indicates the level of initial concentration of 20 mM nitrogen (glutamine or ammonium) in fresh medium.

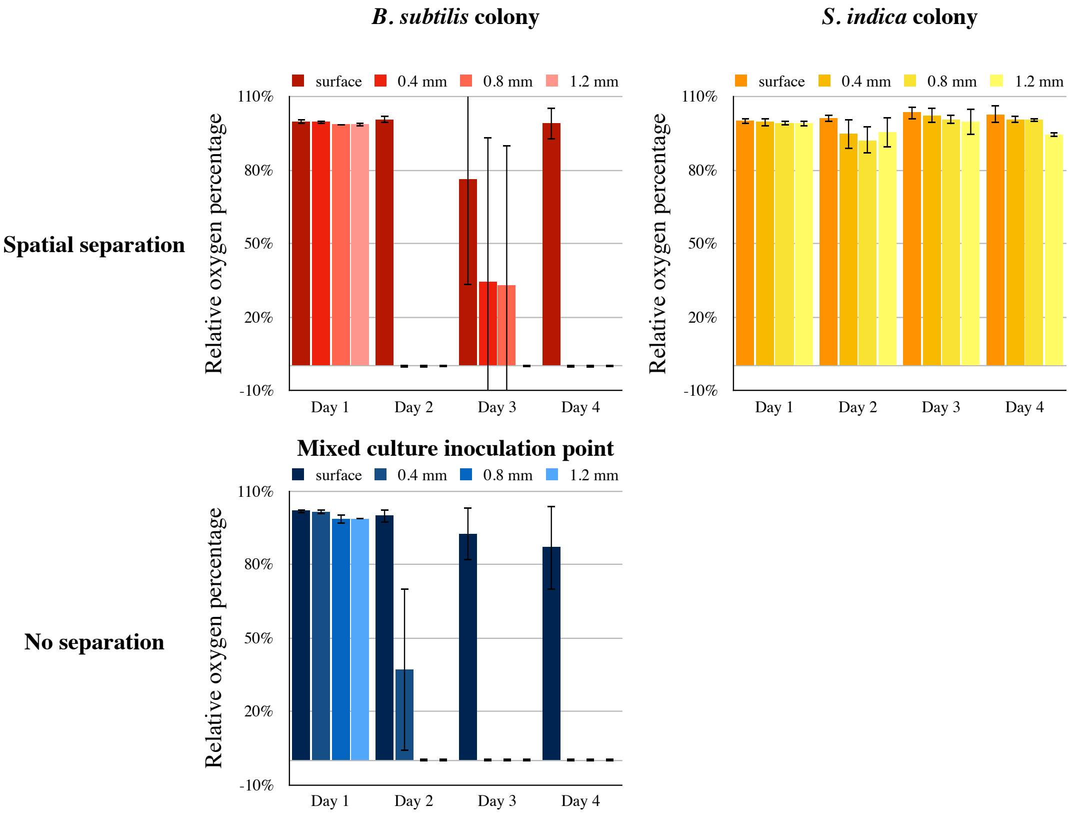

Supplementary Figure 8. Relative oxygen percentage at different depths of plates. "No separation" refers to *B. subtilis* culture and *S. indica spores* being pre-mixed at 1:1 volume ratio, and then inoculated as a single solution. "Spatial separation" refers to approximately 1.5 cm separation of *S. indica* (left) and *B. subtilis* (right) inoculation points. "Surface" indicates the initial measurement point, which was approximately at the surface of a plate/colony. The subsequent measurements are taken at a depth of 0.4, 0.8, and 1.2mm from this initial point, and are labelled with these values on the Figure. Oxygen percentage is shown on the Y-axis, and is normalized against the measurement made at the similar depth on the same plate but on a location without any organism growth. Measurements are from 3 replicate agar plates.

<table>
<tr><th>Gene name</th><th>Function</th><th>Source organism for sequence</th><th>Accession of homologous in S. indica</th></tr>
<tr><td rowspan="2">THI6</td><td>1.hydroxyethylthiazole kinase</td><td rowspan="12">Saccharomyces cerevisiae S288c</td><td rowspan="2">No</td></tr>
<tr><td>2.thiamine-phosphate pyrophosphorylase</td></tr>
<tr><td rowspan="3">THI20</td><td>1.HMP kinase</td><td rowspan="3">No</td></tr>
<tr><td>2.HMP-P kinase</td></tr>
<tr><td>3.Thiaminase II</td></tr>
<tr><td rowspan="2">THI4</td><td>1.Thiamine thiazole synthase</td><td rowspan="2">CCA73069.1</td></tr>
<tr><td>2.Mitochondrial DNA damage tolerance</td></tr>
<tr><td>THI5</td><td>Pyrimidine precursor biosynthesis</td><td>No</td></tr>
<tr><td>THI80</td><td>Thiamine pyrophosphokinase</td><td>CCA69955.1</td></tr>
<tr><td>PHO3</td><td>Thiamin-repressible acid phosphatase</td><td>CCA74294.1</td></tr>
<tr><td>THI7 (THI10)</td><td>Thiamin transporter</td><td>CCA72717.1</td></tr>
</table>

Supplementary Table 1. Results of *S. indica* BLAST analysis, in which *S. indica* genome is searched against thiamine related genes from *S. cerevisiae.*

Supplementary File 1. Timelapse microscopy video showing *S. indica* monoculture on synthetic medium without thiamine.

Supplementary File 2. Timelapse microscopy video showing *S. indica* monoculture on synthetic medium with thiamine.

Supplementary File 3. Timelapse microscopy video showing *S. indica* and *B. subtilis* co-culture on synthetic medium without thiamine.

Supplementary File 4. Timelapse microscopy video showing *S. indica* and *B. subtilis* co-culture on synthetic medium with thiamine.

Supplementary File 5. Table of species for phylogenetic analysis, in which fungi genomes from class *Agaricomycetes,* and fungi available from KEGG database (and listed on the thiamine biosynthesis pathway ortholog table; KEGG pathway 00730), are searched against thiamine related genes from *S. cerevisiae.*
